# Disruptions to School and Home Life Among High School Students During the COVID-19 Pandemic — Adolescent Behaviors and Experiences Survey, United States, January–June 2021

**DOI:** 10.15585/mmwr.su7103a5

**Published:** 2022-04-01

**Authors:** Kathleen H. Krause, Jorge V. Verlenden, Leigh E. Szucs, Elizabeth A. Swedo, Caitlin L. Merlo, Phyllis Holditch Niolon, Zanie C. Leroy, Valerie M. Sims, Xiaoyi Deng, Sarah Lee, Catherine N. Rasberry, J. Michael Underwood

**Affiliations:** ^1^Division of Adolescent and School Health, National Center for HIV, Viral Hepatitis, STD, and TB Prevention, CDC; ^2^Division of Violence Prevention, National Center for Injury Prevention and Control, CDC; ^3^Divison of Population Health, National Center for Chronic Disease Prevention and Health Promotion, CDC; ^4^ICF International, Rockville, Maryland

## Abstract

Youths have experienced disruptions to school and home life since the COVID-19 pandemic began in March 2020. During January–June 2021, CDC conducted the Adolescent Behaviors and Experiences Survey (ABES), an online survey of a probability-based, nationally representative sample of U.S. public- and private-school students in grades 9–12 (N = 7,705). ABES data were used to estimate the prevalence of disruptions and adverse experiences during the pandemic, including parental and personal job loss, homelessness, hunger, emotional or physical abuse by a parent or other adult at home, receipt of telemedicine, and difficulty completing schoolwork. Prevalence estimates are presented for all students and by sex, race and ethnicity, grade, sexual identity, and difficulty completing schoolwork. Since the beginning of the pandemic, more than half of students found it more difficult to complete their schoolwork (66%) and experienced emotional abuse by a parent or other adult in their home (55%). Prevalence of emotional and physical abuse by a parent or other adult in the home was highest among students who identified as gay, lesbian, or bisexual (74% emotional abuse and 20% physical abuse) and those who identified as other or questioning (76% and 13%) compared with students who identified as heterosexual (50% and 10%). Overall, students experienced insecurity via parental job loss (29%), personal job loss (22%), and hunger (24%). Disparities by sex and by race and ethnicity also were noted. Understanding health disparities and student disruptions and adverse experiences as interconnected problems can inform school and community initiatives that promote adolescent health and well-being. With community support to provide coordinated, cross-sector programming, schools can facilitate linkages to services that help students address the adverse experiences that they faced during the ongoing COVID-19 pandemic. Public health and health care professionals, communities, schools, families, and adolescents can use these findings to better understand how students’ lives have been affected during the pandemic and what challenges need to be addressed to promote adolescent health and well-being during and after the pandemic.

## Introduction

Youths have experienced disruptions to school and home life since the COVID-19 pandemic began in March 2020 (*1*). The COVID-19 pandemic has disrupted the lives of adolescents by creating or exacerbating economic, food and nutrition, and housing insecurity as well as experiences of abuse, all of which negatively affect health and well-being (*2*,*3*). Racial and ethnic discrimination is a social determinant of health (*4*), and existing health disparities persisted or worsened during the pandemic. For example, American Indian or Alaska Native, Black, and Hispanic or Latino populations typically experienced higher rates of morbidity and mortality and economic vulnerability compared with the White population before the pandemic and also were more likely than the White population to experience morbidity and mortality from COVID-19 and economic vulnerability during the pandemic (*5*). Adolescents experienced disruptions to education and accessing health care, although schools and health care providers shifted rapidly to virtual platforms and telemedicine to continue providing services (*6*).

To date, no study has assessed national prevalence of disruptions and adverse experiences experienced by high school students during the COVID-19 pandemic. This study addresses this knowledge gap by estimating the prevalence of disruptions and adverse experiences during the pandemic, overall and by sex, race and ethnicity, grade, and sexual identity. Public health and health care professionals, communities, schools, families, and adolescents can use these findings to better understand how students’ lives have been affected during the pandemic and what challenges need to be addressed to promote adolescent health and well-being during and after the pandemic.

## Methods

### Data Source

This report includes data from the Adolescent Behaviors and Experiences Survey (ABES) conducted by CDC during January–June 2021 to assess student behaviors and experiences during the COVID-19 pandemic. ABES was a one-time, probability-based online survey of U.S. high school students. ABES used a stratified, three-stage cluster sample to obtain a nationally representative sample of public- and private-school students in grades 9–12 in the 50 U.S. states and the District of Columbia (N = 7,705). Participation in ABES was voluntary; each school and teacher decided whether students completed the survey during instructional time or on their own time. Additional information about ABES sampling, data collection, response rates, and processing is available in the overview report of this supplement (*7*). The ABES questionnaire, datasets, and documentation are available (https://www.cdc.gov/healthyyouth/data/abes.htm).

### Measures

Students’ self-reported disruptions and adverse experiences were assessed (Table 1), including economic, food and nutrition, and housing insecurity; abuse by a parent or other adult in the home (hereafter referred to as abuse by a parent); receipt of telemedicine; and difficulty completing schoolwork. All questions included the timeframe “During the COVID-19 pandemic,” except for the question about housing insecurity, which asked about experiencing homelessness during the previous 30 days. Demographic variables included sex, race and ethnicity (non-Hispanic American Indian or Alaska Native, non-Hispanic Asian [Asian], non-Hispanic Black [Black], Hispanic or Latino [Hispanic], non-Hispanic persons of multiple races [multiracial], non-Hispanic Native Hawaiian or other Pacific Islander [NH/OPI], and non-Hispanic White [White]), grade (9–12), and sexual identity (gay, lesbian, or bisexual; other or questioning; or heterosexual).

**TABLE 1:** Measures of disruptions and adverse experiences, receipt of telemedicine, and schoolwork difficulty during the COVID-19 pandemic among high school students — Adolescent Behaviors and Experiences Survey, United States, January–June 2021

Student experience	Question	Analytic coding
**Economic insecurity**		
Parent job loss*	During the COVID-19 pandemic, did a parent or other adult in your home lose their job even for a short amount of time?	Yes versus no
Student job loss*	During the COVID-19 pandemic, did you lose your paying job even for a short amount of time?	Yes versus no
**Food and nutrition insecurity**		
Hunger	During the COVID-19 pandemic, how often did you go hungry because there was not enough food in your home?	Yes (rarely, sometimes, most of the time, or always) versus no (never)
**Housing insecurity**		
Homelessness	During the past 30 days, where did you usually sleep?	Yes (in the home of a friend, family member, or other person because I had to leave my home; my parent or guardian cannot afford housing; in a shelter or emergency housing; in a motel or hotel; in a car, park, campground, or other public place; or I do not have a usual place to sleep) versus no (in my parent's or guardian's home)
**Abuse by a parent**		
Emotional abuse	During the COVID-19 pandemic, how often did a parent or other adult in your home swear at you, insult you, or put you down?	Yes (rarely, sometimes, most of the time, or always) versus no (never)
Physical abuse	During the COVID-19 pandemic, how often did a parent or other adult in your home hit, beat, kick, or physically hurt you in any way?	Yes (rarely, sometimes, most of the time, or always) versus no (never)
**Received telemedicine**		
Care from a doctor or nurse	During the COVID-19 pandemic, did you get medical care from a doctor or nurse using a computer, phone, or other device (also called telemedicine)?	Yes versus no
Mental health or drug and alcohol counseling	During the COVID-19 pandemic, did you get mental health care, including treatment or counseling for your use of alcohol or drugs using a computer, phone, or other device (also called telemedicine)?	Yes versus no
**Schooling**		
Schoolwork difficulty	Do you agree or disagree that doing your schoolwork was more difficult during the COVID-19 pandemic than before the pandemic started?	Yes (strongly agree, or agree) versus no (not sure, disagree, or strongly disagree)

*The denominator includes only those who had jobs prior to the beginning of the pandemic.

### Analysis

Weighted prevalence estimates and 95% CIs were calculated for disruptions and adverse experiences; estimates were calculated among all students and by demographic characteristics. Bivariate associations between disruptions and adverse experiences and difficulty completing schoolwork are presented. Pairwise *t*-tests were used to compare prevalence estimates between groups. Estimates were suppressed when n<30; consequently, all results for NH/OPI students were suppressed. Statistical significance was assessed at p<0.05; only significant results are presented. Analyses were completed using SUDAAN (version 11.0.1; RTI International) to account for the complex survey design and weighting.

## Results

More than one fourth of adolescents experienced a parent losing a job (28.5%), and nearly one fourth experienced their own job loss (22.3%) or hunger (23.8%) during the COVID-19 pandemic (Table 2). Some experienced homelessness (2.0%). Over half of adolescents experienced emotional abuse by a parent (55.1%), and more than one in 10 experienced physical abuse by a parent (11.3%). Approximately one fourth of students received telemedicine from a doctor or nurse (25.8%), and some received telemedicine for mental health or drug and alcohol counseling (8.5%). Two thirds of students had difficulty completing their schoolwork since the start of the pandemic (66.6%).

**TABLE 2:** Percentage of economic, food and nutrition, and housing insecurity, abuse by a parent, receipt of telemedicine, and schoolwork difficulty among high school students during COVID-19 pandemic, by sex, race and ethnicity, and grade — Adolescent Behaviors and Experiences Survey, United States, January–June 2021

Characteristic	Economic insecurity		Food and nutrition insecurity	Housing insecurity	Abuse by a parent		Received telemedicine		Schooling
	Parent job loss	Student job loss	Hunger	Homelessness	Emotional abuse	Physical abuse	Care from a doctor or nurse	Mental health or drug and alcohol counseling	Schoolwork difficulty
	%* (95% CI)	%* (95% CI)	%* (95% CI)	%* (95% CI)	%* (95% CI)*	%* (95% CI)	%* (95% CI)	%* (95% CI)	%* (95% CI)
**Sex**									
Female	31.3 (28.9–33.9)	25.5 (22.3–29.0)	24.9 (22.1–28.0)	1.1 (0.8–1.4)	62.8 (59.5–66.1)	11.6 (10.1–13.2)	29.8 (27.1–32.7)	10.1 (8.5–11.9)	69.1 (66.0–72.0)
Male	25.6 (23.0–28.4)^†^	19.0 (15.7–22.8)^†^	22.7 (20.3–25.2)	3.0 (2.1–4.2)^†^	46.8 (44.2–49.4)^†^	10.9 (9.7–12.2)	21.7 (19.7–23.9)^†^	6.5 (5.5–7.7)^†^	64.1 (62.0–66.1)^†^
**Race and ethnicity**									
AI/AN, non-Hispanic	15.7 (9.1–25.5)	—^§^	31.2 (19.2–46.4)	1.9 (0.8–4.7)	54.9 (47.7–62.0)	12.5 (8.3–18.5)	24.1 (15.8–34.7)	7.0 (2.5–18.1)	72.4 (64.2–79.3)
Asian, non-Hispanic	37.1 (29.3–45.7)^¶^	18.7 (12.5–27)	28.3 (22.5–34.9)	2.2 (0.7–7.4)	59.2 (54.5–63.7)	12.9 (8.9–18.5)	24.7 (15.2–37.6)	3.9 (2.3–6.7)	61.7 (51.5–70.9)
Black, non-Hispanic	24.9 (21.4–28.7)^¶,^**	23.6 (18.6–29.5)	32.0 (28.4–35.7)	2.5 (1.6–3.8)	49.6 (44.0–55.2)**	15.0 (11.6–19.1)	21.1 (17.8–24.9)	6.3 (4.5–8.7)	67.7 (63.3–71.7)
Hispanic or Latino	38.0 (33.9–42.2)^¶,††^	21.8 (17.0–27.5)	28.2 (24.1–32.7)	1.7 (1.1–2.5)	52.5 (48.0–56.9)	11.2 (9.4–13.3)	22.3 (19.1–25.8)	5.4 (4.2–7.0)	69.4 (65.4–73.1)
Multiracial, non-Hispanic	25.4 (18.8–33.4)^¶, ^**^,§§^	25.1 (17.4–34.7)	29.5 (23.4–36.5)	1.1 (0.5–2.5) ^††^	65.5 (59.7–70.8) ^¶,††,§§^	13.4 (10.6–16.9)	29.7 (24.2–35.8)^††,§§^	15.0 (10.8–20.5)^¶,^**^,††,§§^	67.3 (60.5–73.5)
White, non-Hispanic	24.4 (22.0–27.1)^¶,^**^,§§^	22.2 (19.0–25.8)	18.5 (16.5–20.6)**^,††,§§,¶¶^	2.1 (1.5–2.9 )	56.4 (52.3–60.3)^††,¶¶^	9.8 (8.6–11.1)^††^	28.8 (26.2–31.6)^††,§§^	10.2 (8.7–11.9)**^,††,§§^	65.5 (63.1–67.8)
**Grade**									
9	29.1 (24.7–33.8)	12.6 (9.5–16.6)	24.9 (21.3–28.8)	1.9 (1.3–2.7)	58.0 (54.1–61.8)	14.3 (12.1–16.7)	23.9 (21.4–26.5)	8.0 (6.4–9.9)	66.8 (63.5–69.9)
10	29.0 (26.3–31.8)	17.2 (13.9–21.0)	23.9 (20.4–27.9)	2.3 (1.4–3.6)	54.4 (51.5–57.3)	11.9 (10.0–14.0)	25.7 (22.8–28.8)	8.9 (7.4–10.8)	67.1 (63.9–70.2)
11	27.4 (24.1–31.0)	18.6 (14.8–23.0)***	23.1 (20.3–26.2)	1.6 (0.9–2.8)	53.9 (49.4–58.4)	10.6 (8.4–13.3)***	25.8 (23.1–28.7)	8.1 (6.4–10.3)	65.6 (61.9–69.2)
12	28.3 (25.0–31.8)	33.6 (29.6–37.9)***^,†††,§§§^	23.3 (20.2–26.7)	2.3 (1.4–3.6)	53.8 (49.9–57.6)***	7.7 (6.4–9.3)***^,†††^	28.3 (24.4–32.5)***	9.0 (7.0–11.4)	66.9 (62.6–70.9)
**Total**	**28.5 (26.2–30.9)**	**22.3 (19.8–24.9)**	**23.8 (21.6–26.3)**	**2.0 (1.5–2.6)**	**55.1 (52.3–57.8)**	**11.3 (10.2–12.4)**	**25.8 (23.8–28.0)**	**8.5 (7.4–9.6)**	**66.6 (64.5–68.6)**

**Abbreviation: **AI/AN = American Indian or Alaska Native.

* Weighted estimate.

^†^ Pairwise *t*-test significantly different from female students (p<0.05).

^§^ Dash indicates that results are suppressed because n<30.

^¶^ Pairwise *t*-test significantly different from non-Hispanic AI/AN students (p<0.05).

** Pairwise *t*-test significantly different from non-Hispanic Asian students (p<0.05).

^††^ Pairwise *t*-test significantly different from non-Hispanic Black students (p<0.05).

^§§^ Pairwise *t*-test significantly different from Hispanic or Latino students (p<0.05).

^¶¶^ Pairwise *t*-test significantly different from non-Hispanic multiracial students (p<0.05).

*** Pairwise *t*-test significantly different from 9th-grade students (p<0.05).

^†††^ Pairwise *t*-test significantly different from 10th-grade students (p<0.05).

^§§§^ Pairwise *t*-test significantly different from 11th-grade students (p<0.05).

Student disruptions and adverse experiences differed by sex and race and ethnicity. Female students experienced a higher prevalence of parental and personal job loss (31.3% and 25.5%), emotional abuse by a parent (62.8%), and difficulty completing schoolwork (69.1%) compared with male students; whereas males experienced homelessness (3.0%) more often. The prevalence of parental job loss was higher among Asian (37.1%) and Hispanic or Latino (38.0%) students compared with all other racial and ethnic groups. Black students experienced the highest prevalence of hunger (32.0%); this estimate is similar to other students of color. White students had the lowest prevalence of experiencing hunger (18.5%), which differed from most other racial and ethnic groups. Multiracial students reported the highest prevalence of emotional abuse by a parent (65.5%), which differed from most other racial and ethnic groups. Black students experienced the highest prevalence of physical abuse by a parent (15.0%); this estimate did not differ from most other students of color, but it was higher than the prevalence of physical abuse experienced by White students (9.8%). Students who reported difficulty completing their schoolwork reported a higher percentage of parental job loss, hunger, or emotional abuse by a parent compared with students who did not have difficulty completing their schoolwork (Table 3).

**TABLE 3:** Percentage of economic, food and nutrition, and housing insecurity, abuse by a parent, and receipt of telemedicine among high school students during COVID-19 pandemic, by schoolwork difficulty — Adolescent Behaviors and Experiences Survey, United States, January–June 2021

Experience	Experienced schoolwork difficulty	
	Yes	No
	%* (95% CI)	%* (95% CI)
**Economic insecurity**		
Parent job loss	30.9 (28.4–33.4)	23.9 (20.9–27.2)^†^
Student job loss	23.0 (20.5–25.8)	20.7 (17.2–24.7)
**Food and nutrition insecurity**		
Hunger	25.5 (22.9–28.2)	20.6 (18.1–23.4)^†^
**Housing insecurity**		
Homelessness	1.9 (1.4–2.6)	2.2 (1.5–3.1)
**Abuse by a parent**		
Emotional abuse	58.3 (55.5–61.2)	48.7 (44.9–52.5)^†^
Physical abuse	11.3 (10.1–12.6)	11.1 (9.1–13.5)
**Received telemedicine**		
Care from a doctor or nurse	26.3 (24.1–28.7)	24.7 (22.0–27.6)
Mental health or drug and alcohol counseling	8.1 (6.9–9.5)	9.2 (7.6–11.2)

* Weighted estimate.

^†^ Pairwise *t*-test significantly different from students who responded "yes" (p<0.05).

Students who identified as gay, lesbian, or bisexual and those who identified as other or questioning experienced a higher prevalence of parental job loss (34.9% and 34.9%, respectively), hunger (34.0% and 32.5%, respectively), and emotional abuse by a parent (74.4% and 75.9%, respectively) compared with heterosexual students (Figure) (Supplementary Table, https://stacks.cdc.gov/view/cdc/114936). Gay, lesbian, or bisexual students experienced a higher prevalence of physical abuse by a parent and difficulty completing their schoolwork (19.7% and 74.4%, respectively) than students who identified as other or questioning (13.4% and 63.8%, respectively) or as heterosexual (9.5% and 65.9%, respectively).

**FIGURE:**
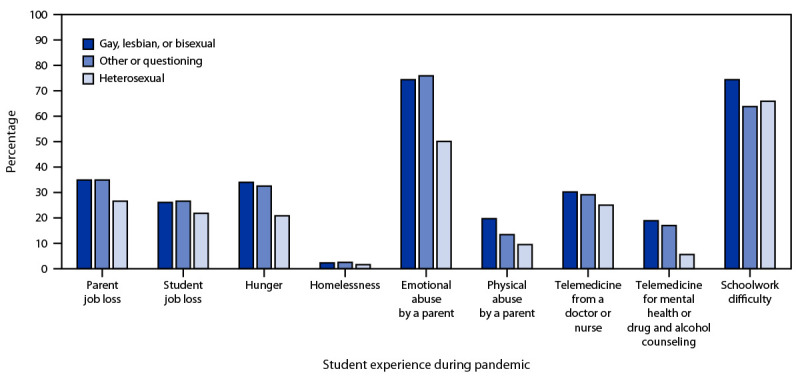
Percentage* of parent job loss,^†,§^ student job loss, hunger,^†,§^ homelessness, emotional abuse by a parent,^†,§^ physical abuse by a parent^,†,§,¶^ receipt of telemedicine by a nurse or doctor,^†,§^ receipt of telemedicine for mental health or drug and alcohol counseling,^†,§^ and schoolwork difficulty^†,¶^ among high school students during the COVID-19 pandemic, by sexual identity — Adolescent Behaviors and Experiences Survey, United States, January–June 2021 * Weighted estimate. ^†^ Pairwise *t*-test heterosexual students significantly different from gay, lesbian, or bisexual students (p<0.05). ^§^ Pairwise *t*-test heterosexual students significantly different from other or questioning students (p<0.05). ^¶^ Pairwise *t*-test other or questioning students significantly different from gay, lesbian, or bisexual students (p<0.05).

## Discussion

During January–June 2021, approximately half of high school students in the United States reported emotional abuse by a parent or reported difficulty completing their schoolwork since the COVID-19 pandemic began in March 2020. In addition, nearly one in four students reported experiencing hunger or economic insecurity and one in 10 students reported physical abuse by a parent. These findings indicate that adolescents have encountered disruptions and adverse experiences during the pandemic that might impact their immediate and long-term health and well-being.

The finding that more than half of adolescents reported emotional abuse and one in 10 reported physical abuse by a parent or other adult in the home during the pandemic is a public health concern; comparatively, a nationally representative sample from the National Survey of Children’s Exposure to Violence (NSCEV)reported a lower proportion of children aged 14–17 years (13.9% for past-year emotional abuse and 5.5% for past-year physical abuse by a caregiver) (*8*). Although these differences might be attributable in part to variations in sampling frame, methodology (e.g., NSCEV is not school based and is administered by telephone) and question wording, the high prevalence of self-reported emotional and physical abuse during the pandemic highlights that increased stress contributes to violence. The situation is further complicated by the fact that school closings because of COVID-19 have resulted in students’ decreased contact with mandated reporters (*9*); therefore, the self-reported data in this report are critically important to elucidate the occurrence of child abuse during the pandemic and underscores the need for enhanced violence surveillance and prevention strategies during public health emergencies.

Disparities in experiences of disruption and adversity were observed by sexual identity, race and ethnicity, and sex. Students identifying as gay, lesbian, or bisexual, other or questioning; students of color;,and female students more commonly had disruptions and adverse experiences compared with heterosexual, White, and male students, respectively. Among any demographic grouping, youths who identified as gay, lesbian, or bisexual and other or questioning experienced the highest levels of emotional and physical abuse by a parent. Per analyzed chat transcripts from national online LGBTQ+ support groups for youth, adolescents identifying as lesbian, gay, bisexual, or questioning have been struggling with isolation during the COVID-19 pandemic and coping with family dynamics described as “unsupportive” and “homophobic” (*10*). Disparities based on race and ethnicity and sex have been documented throughout the pandemic. Previous research shows that during the pandemic, Black and Hispanic or Latino students were more likely to be in households experiencing food and nutrition insecurity, difficulty paying rent, and difficulty affording household expenses compared with White students (*11*), and that approximately two thirds of female adolescents reported an increase in household chores during the pandemic compared with less than half (43%) of the boys, and more girls (20%) compared with boys (10%) reported having too many chores to do to be able to learn (*12*).

Many student disruptions and adverse experiences in this report are interconnected with the social determinants of health. Previous research shows that disparities based on race and ethnicity and sex existed among persons who experienced economic, food and nutrition, or housing insecurity before the pandemic, and these persons had a greater likelihood of experiencing these insecurities during the pandemic (*13*). In addition, financial and social stressors of the COVID-19 pandemic have been documented as risk factors for increased child abuse (*9*). Finally, the bivariate analysis provides evidence that these experiences are interconnected; students who had difficulty completing their schoolwork experienced higher levels of parental job loss, food and nutrition insecurity, and emotional abuse.

One in four students reported using telemedicine to access care from a doctor or nurse and less than one in 10 reported using telemedicine to access mental health or drug and alcohol counseling, with differences by sex and race and ethnicity; White and multiracial students and female students using telemedicine more than other groups. Given the paucity of data on adolescent use of telemedicine, the context for the telemedicine findings of this report remains unclear. Telemedicine might serve as an alternative access point for adolescents seeking essential health services that might address disruptions and adverse experiences, but data describing adolescents’ prepandemic telemedicine use are lacking. A study using data from four major U.S. telehealth providers found that use of telemedicine decreased slightly among youths aged 5–17 years at the start of the pandemic in early 2020 (8.6%) compared with early 2019 (10.0%) (*6*), which reflects a lower use than what was found in this report. Future studies could help researchers better understand the range of telemedicine services received and quality of care.

Two thirds of adolescents had difficulty completing their schoolwork since the beginning of the pandemic. These findings are consistent with previous research, which indicates that throughout the COVID-19 pandemic, adolescents have had difficulty transitioning to virtual learning, reporting inconsistencies in school coursework expectations, and confusion about complex and complicated assignments (*14*). Students who had difficulty completing their schoolwork experienced higher levels of emotional abuse by a parent, parental job loss, and hunger. These disruptions and adverse experiences threaten adolescents’ health and safety in addition to acting as barriers to learning. Learning is fostered in environments where students’ basic needs are met and where students feel safe, supported, challenged, and engaged (*15*). Before the pandemic, schools offered essential health services and social supports, such as school meals, chronic disease management, and mental health counseling; however, the pandemic has challenged the ability of schools to meet students’ evolving academic and health needs (*16*).

Schools offer an important pathway to help address the needs of students, but they rely on coordinated efforts across sectors to meet these needs. Prioritization of school health programs and services within schools, in collaboration with families and communities, will be critical to address disruptions to student life and other related effects of the pandemic (*17*). For example, during the pandemic, the U.S. Department of Agriculture issued multiple waivers that permitted schools flexibility in distributing free meals to school-aged youths, regardless of family income level, through June 2022 (*18*). In addition to traditional meal service in schools, meals are also being distributed in alternative locations, including along school bus routes and in school parking lots and churches (*18*). Coordinated, cross-sector programs and services like these are important for providing continued support for students in their lives both inside and outside of school.

## Limitations

General limitations to ABES are outlined in the overview report in this supplement (*7*). The findings in this report are subject to at least three specific limitations First, causality or directionality of observed association cannot be determined; although the questions about disruption and adversity ask students about what happened to them during the pandemic (e.g., temporaility associated), it cannot be ascertained that the pandemic caused these student experiences. Second, the telemedicine measures should be interpreted with caution given the unknown context for students’ prepandemic use of telemedicine services. Although most students did not receive telemedicine care from a doctor or nurse since the beginning of the pandemic, students might have accessed in-person health care or might not have needed well-child or other health care visits. In addition, without knowing who provided the care or for which reason, the receipt of telemedicine for mental health or drug and alcohol counseling might not align with students’ needed access to care. Finally, the prevalence of food and nutrition insecurity might have been misclassified because self-reported hunger was used as a proxy measure and this measure has not been validated. In addition, other factors are associated with food and nutrition insecurity (e.g., reducing the size of a meal or the variety of foods consumed) (*19*).

## Conclusion

Since the beginning of the COVID-19 pandemic in March 2020, many high school students have experienced hunger and economic insecurity. More than half of students have experienced emotional abuse by a parent and have had difficulty completing their schoolwork. Approximately 10% reported physical abuse by a parent. Disparities by sex, race and ethnicity, and sexual identity highlight the importance of strategies to increase health equity in these domains. Understanding health disparities and student experiences of disruptions and adverse experienes as interconnected problems can inform school and community initiatives that promote adolescent health and well-being. With community support to provide coordinated, cross-sector programming, schools can serve as the setting to facilitate linkages to services that help address the adverse experiences that students have faced during the pandemic. Public health and health care professionals, communities, schools, families, and adolescents can use these findings to better understand how students’ lives have been affected during the pandemic and what challenges need to be addressed to promote adolescent health and well-being during and after the pandemic.
